# Long-term impacts of prostate cancer treatments on sexual function and psychological well-being: A comprehensive narrative review

**DOI:** 10.1192/j.eurpsy.2025.1779

**Published:** 2025-08-26

**Authors:** R. Arif, H. Hami, S. Sennani, S. Irnat, A. Mokhtari, S. Jaouhar, A. Soulaymani, J. Bouzid, F. Hadrya

**Affiliations:** 1 University Hassan First of Settat, Higher Institute of Health Sciences, Health Sciences and Technologies Laboratory, Settat; 2 Laboratory of Biology and Health, Faculty of Science, Ibn Tofail University, Kenitra; 3 Higher Institute of Nursing and Technical Health Professions, Fez, Meknes Annex, Morocco

## Abstract

**Introduction:**

Sexual dysfunction, which is frequently observed in cancer patients, especially those undergoing prostate cancer treatments, has significant implications for both psychological well-being and intimate relationships. This review consolidates the current understanding of treatment impacts on this patient group.

**Objectives:**

This study examines the prevalence and psychological impact of sexual dysfunction among prostate cancer survivors following treatment.

**Methods:**

A narrative review was conducted, analyzing data from six observational studies involving 7,146 participants and one meta-analysis published between 2015 and 2024. These studies focused on elucidating the relationship between sexual dysfunction and psychological well-being post-treatment using databases such as Scopus, PubMed, and Web of Science.

**Results:**

All main prostate cancer treatments (surgery, radiotherapy, and hormonal therapy) are associated with substantial risks of sexual and erectile dysfunction, as well as depressive symptoms. Notably, radical prostatectomy significantly reduces sexual function, with about one-third of patients experiencing depressive symptoms post-surgery. Additionally, within a year of undergoing radical prostatectomy, approximately 90% of patients experience moderate to severe erectile dysfunction. The associated depressive symptoms largely stem from diminished sexual activity and dissatisfaction stemming from disrupted sexual normalcy. Anti-androgen therapies also progressively worsen sexual function, impacting perceived masculinity and contributing to psychological distress. Notably, older men generally adapt to these changes more adeptly than younger men.

**Conclusions:**

Persistent erectile dysfunction is a major, detrimental side effect for prostate cancer survivors, profoundly impacting their quality of life. Enhanced education for patients and their partners about potential sexual side effects, along with improved awareness and preparedness strategies, are essential to alleviate the psychological burdens stemming from post-treatment sexual dysfunctions.

**Disclosure of Interest:**

None Declared

